# Pilot study: Effects of drinking hydrogen-rich water on muscle fatigue caused by acute exercise in elite athletes

**DOI:** 10.1186/2045-9912-2-12

**Published:** 2012-07-12

**Authors:** Kosuke Aoki, Atsunori Nakao, Takako Adachi, Yasushi Matsui, Shumpei Miyakawa

**Affiliations:** 1 Doctoral Program in Sports Medicine, Graduate School of Comprehensive Human Sciences, University of Tsukuba, Ibaraki, Japan; 2 Department of Emergency and Critical Care Medicine, Hyogo College of Medicine, 1-1, Mukogawa-cho, Nishinomiya, Hyogo, 663-8501, Japan

## Abstract

**Background:**

Muscle contraction during short intervals of intense exercise causes oxidative stress, which can play a role in the development of overtraining symptoms, including increased fatigue, resulting in muscle microinjury or inflammation. Recently it has been said that hydrogen can function as antioxidant, so we investigated the effect of hydrogen-rich water (HW) on oxidative stress and muscle fatigue in response to acute exercise.

**Methods:**

Ten male soccer players aged 20.9 ± 1.3 years old were subjected to exercise tests and blood sampling. Each subject was examined twice in a crossover double-blind manner; they were given either HW or placebo water (PW) for one week intervals. Subjects were requested to use a cycle ergometer at a 75 % maximal oxygen uptake (VO_2_) for 30 min, followed by measurement of peak torque and muscle activity throughout 100 repetitions of maximal isokinetic knee extension. Oxidative stress markers and creatine kinase in the peripheral blood were sequentially measured.

**Results:**

Although acute exercise resulted in an increase in blood lactate levels in the subjects given PW, oral intake of HW prevented an elevation of blood lactate during heavy exercise. Peak torque of PW significantly decreased during maximal isokinetic knee extension, suggesting muscle fatigue, but peak torque of HW didn’t decrease at early phase. There was no significant change in blood oxidative injury markers (d-ROMs and BAP) or creatine kinease after exercise.

**Conclusion:**

Adequate hydration with hydrogen-rich water pre-exercise reduced blood lactate levels and improved exercise-induced decline of muscle function. Although further studies to elucidate the exact mechanisms and the benefits are needed to be confirmed in larger series of studies, these preliminary results may suggest that HW may be suitable hydration for athletes.

## Introduction

Since energy demands and oxygen consumption increase during supermaximal exercise, such as intermittent running, sprints, and jumps, production of reactive oxygen species (ROS) and reactive nitrogen species (RNS) also increase, threatening to disturb redox balance and cause oxidative stress. During normal conditions, ROS and RNS are generated at a low rate and subsequently eliminated by the antioxidant systems. However, a greatly increased rate of ROS production may exceed the capacity of the cellular defense system. Consequently, substantial free radicals’ attack on cell membranes may lead to a loss of cell viability and to cell necrosis and could initiate the skeletal muscle damage and inflammation caused by exhaustive exercise [1-3]. Although well-trained athletes suffer from less oxidative stress reduction because their antioxidant systems adapt, accumulation of intense exercise can provoke an increase in oxidative stress [4]. To mitigate oxidative stress-induced adverse events during sports, antioxidant supplementation among athletes has been well documented. Although results of these studies are often contradictory depending on the antioxidant compounds and quantity, some studies demonstrate the beneficial effects of antioxidants on muscle fatigue or performance [5,6].

Recently, the beneficial effects of hydrogen-rich water (HW) have been described in experimental and clinical disease conditions [7,8]. Although research on the health benefits of HW is limited and there is scant data on long-term effects, pilot studies on humans suggest that consuming HW may help prevent metabolic syndrome [9], diabetes mellitus [10], and cancer patients’ side effects with radiotherapy [11]. Since hydrogen is known to scavenge toxic ROS [12] and induce a number of antioxidant proteins [13,14], we hypothesized that drinking HW may be beneficial for athletes in reducing oxidative stress-induced muscle fatigue following acute exercise. In this study, we evaluated the efficacy of hydrogen-rich water on healthy subjects by measuring muscle fatigue and blood lactate levels after exercise. Although further studies are needed to elucidate the exact mechanisms and benefits, this report suggests that hydrogen-rich water might be an appropriate hydration fluid for athletes.

## Methods

### Subjects

Ten male soccer players aged 20.9 ± 1.3 years old were subjected to exercise tests and blood sampling. None of the subjects were smokers or were taking any supplements/medicines. Each subject provided written informed consent before participation in accordance with the University of Tsukuba’s Human Research Ethics Committee. Physical characteristics of the subjects are shown in Table 1. All the players were involved in daily training sessions except the day of experiment.

**Table 1:** Subjects’ Physical Characteristics (n = 10)

Variable	Value
Age (year)	20.9 ± 1.3
Height (cm)	172.0 ± 3.8
Body weight (kg)	67.1 ± 5.2
BMI (kg/m^2^)	22.8 ± 1.4
VO_2_max (ml/kg/min)	53.2 ± 4.9

BMI: body mass index, VO_2_max: maximal oxygen uptake.

### Generation of hydrogen-rich water

A plastic shelled product consisting of metallic magnesium (99.9 % pure) and natural stones in polypropylene containers combined with ceramics (Doctor SUISOSUI®, Friendear, Tokyo, Japan) was used to produce hydrogen. The product was capable of generating hydrogen when placed in drinking water via the following chemical reaction: Mg + 2H_2_O → Mg (OH)_2_ + H_2_. The magnesium stick or a placebo (a casting-only stick without magnesium) was immersed in mineral water (Volvic®, Kirin Inc., Tokyo) for 24 hours prior to drinking. The final hydrogen concentrations of the placebo water (PW) and hydrogen-rich water (HW) were 0 and 0.92 ~ 1.02 mM, respectively [9,11]. Each subject was examined twice in a crossover double-blind manner, given either HW or PW for one week intervals.

### Dose and mode of administration of hydrogen-rich water

Subjects were provided with three 500 ml bottles of drinking water and instructed to place two magnesium sticks in each bottle 24 hours prior to drinking. Participants were asked to drink one bottle at 10:00 PM of the day before the test, one at 5:00 AM, and one at 6:20 AM on the day of examination. In summary, subjects consumed 1,500 ml of HW or PW.

### Protocol

The research protocol started at 6:00 AM. Subjects were given meals between 9:00 PM and 10:00 PM the day before experiments, and fasted overnight. No breakfast was given on the day of the experiments. The subjects were first required to rest in a sitting position for 30 minutes. The exercise test consisted of the following: 1) Maximal progressive exercise test to define maximal oxygen uptake (VO_2_max); 2) cycling an ergometer for 30 minutes at approximately 75 % VO_2_max (Exercise-1); and 3) Running 100 maximal isokinetic knee extensions at 90 ° sec^-1^ (Exercise-2). Blood samples were collected from an antecubital vein just before Exercise-1 (6:30 AM), immediately after Exercise-1 (7:15 AM), immediately after Exercise-2 (7:30 AM), 30 minutes after Exercise-2 (8:00 AM) and 60 minutes after Exercise-2 (8:30 AM).

### Maximal progressive exercise test

First, to define maximal oxygen uptake (VO_2_max), the subjects were subjected to a maximal progressive exercise test on a bicycle ergometer (232CL, Conbiwellness, Tokyo). The test consisted of a continuous step test beginning at a 30 W load, and increasing by 20 W every minute until exhaustion. The subjects were instructed to ride at 50 rpm/min. Pulmonary gas exchange values were measured using an exhaled gas sensor (AE280S, Minato Medical®, Osaka, Japan) via a breath-by-breath system, and the mean values were calculated every 30 seconds for analysis. We determined that VO_2_max was reached when the oxygen consumption reached its plateau [15].

#### Fixed-load cycling at 75 % (high intensity) of VO_2_ max

Before the test started, the subjects rested for two minutes. After warming up at a load of 50 W for one minute, the subjects were instructed to ride at submaximal levels for 30 minutes. Pulmonary gas exchange values were monitored to maintain VO_2_max at approximately 75 %. During the experiments, the subjects were frequently verbally instructed to control the range of motion to maintain VO_2_max at approximately 75 %.

#### Maximal isokinetic knee extensions

A calibrated Biodex System 3 isokinetic device (Biodex Medical Systems, New York, USA) was used to measure peak torque (PT) and knee-joint position throughout 100 repetitions of maximal isokinetic knee extension. During testing, each subject was seated on the Biodex system 3 with 90° hip flexion, and restraining straps were placed across the waist and chest in addition to a rigid sternal stabilizer. The dynamometer was motor driven at a constant velocity of 90°/sec. Each subject performed a series of 100 isokinetic contractions using the knee extensors of the right leg from 90° of flexion to 0° (full extension). As the arm of the dynamometer moved up from 90° to 0°, subjects were encouraged to perform maximally for each contraction throughout the full range of motion. Subjects relaxed as the dynamometer arm moved back to 90°. Each contraction and relaxation period lasted one second and the total length of the contraction cycle was thus two seconds. All subjects were able to complete the full 100 contractions.

#### Measurement of muscle fatigue

To measure muscle fatigue, the widely used First Fourier transform technique (FFT) is utilized to analyze mean frequency of surface electromyogram (EMG) [16]. EMG signals were obtained from the rectus femoris muscle via electrodes connected to a 4-channel frequency-modulation transmitter (Nihon Kohden, Tokyo, Japan). All data were stored and analyzed using the FFT functions in Acknowledge 3.7.5 software (BIOPAC SYSTEM, Santa Barbara, USA). Mean power frequency (MPF) and median power frequency (MDF) were calculated as previously described [17]. MPF shift of the EMG signal toward lower frequencies has been extensively used in static contractions to indicate the development of peripheral fatigue.

#### Blood test

Blood lactate levels were determined using a commercially available Lactate Pro LT17170 kit (Arkray, Inc., Kyoto, Japan). The concentrations of derivatives of reactive oxidative metabolites (dROMs) and biological antioxidant power (BAP) in the peripheral blood were assessed using a Free Radical Analytical System (FRAS4; Wismerll, Tokyo, Japan). Laboratory tests for creatine kinase (CK) were conducted using standardized procedures at Kotobiken Medical Laboratory Services (Tokyo, Japan).

#### Statistical analysis

Repeated analysis of variance (ANOVA) tests were used to compare pre- and post-exercise measurements. The F-test with Bonferroni *post hoc* group comparisons was performed where appropriate. Probability values less than 0.05 were considered to be statistically significant. SPSS 18.0 was used to perform the statistical analysis. Since the experiment was planned to have a 90 % power of achieving significance at the 5 % level, the sample size in this model is calculated to be between 8.91 and 9.25 (90 % power and 5 % significance level) in blood lactate levels based on our previous experiences. Therefore, we assumed the sample size would be appropriate for accumulation of preliminary data.

## Results

### Blood analysis for lactic acid, d-ROMs, BAP and CK

**Figure 1:**
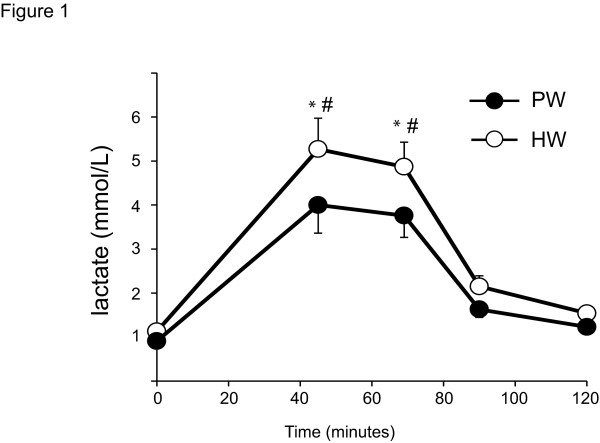
**Sequential changes of blood lactate levels during exercise.** Blood lactate levels in the athletes given PW significantly increased immediately after exercise compared to the levels at pre-exercise. HW significantly reduced blood lactate levels post exercise using bicycle ergometer. (*p < 0.05 vs. time 0. #p < 0.05 vs HW, N = 10).

As shown in Table 2, blood d-ROMs BAP and CK levels increased after exercise in subjects in both groups treated with PW and HW. However, there was no statistical difference between the groups. Eventhough the blood lactate level were significantly increased in both HW and PW at 45 and 60 min after exercise, these levels were comparably and significantly lower in the HW than in the PW group (Figure 1).

**Table 2:** Changes in Blood Levels

		0 min	45 min	60 min	90 min	120 min
d-ROMs (U.CARR)	PW	269.0 ± 50.8	285.7 ± 52.3*	287.0 ± 56.9*	274.2 ± 50.2	280.0 ± 47.6
	HW	281.3 ± 61.8	303.5 ± 46.3*	308.6 ± 56.1*	296.1 ± 57.9	307.0 ± 45.8
BAP (μmol/L)	PW	2347.3 ± 155.8	2648.9 ± 96.5*	2632.8 ± 146.4*	2349.6 ± 152.0	2321.8 ± 196.9
	HW	2336.7 ± 123.1	2659.1 ± 102.1*	2664.6 ± 201.0*	2299.8 ± 104.6	2356.4 ± 143.7
CK (IU/L)	PW	247.0 ± 105.1	296.5 ± 119.9*	300.9 ± 127.7*	264.7 ± 113.3*	256.3 ± 111.7
	HW	407.4 ± 269.9	483.2 ± 314.0*	478.1 ± 314.5*	428.2 ± 282.0	353.7 ± 264.6

Data were shown as mean ± standard deviation (SD). *p < 0.05 vs 0 min.

### Maximal knee extension exercise

**Figure 2:**
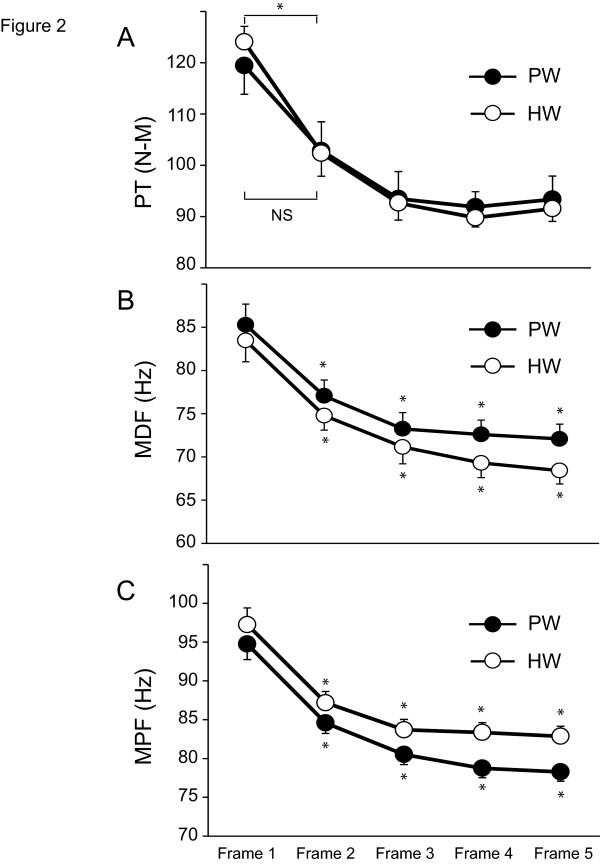
**(A) Changes in peak torque (PT) every 20 repetitions (rep = 1 frame) during 100 maximum isokinetic knee extensions.** PT of the subjects treated with PW significantly decreased during the initial 40-60 contractions by approximately 20-25 % of the initial values, followed by a phase with little change. On the other hand, there was no statistical difference between Frame 1 and Frame 2 in HW, indicating that HW prevented the decreasing the peak torque during the first 2 Frames. HW, Hydrogen rich water; PW, Placebo water. (*p < 0.05 vs Frame 1, N = 10). **(B) Changes in median frequency (MDF) every 20 repetitions (rep = 1 Frame) during 100 maximum isokinetic knee extensions.** Although exercise significantly reduced MDF values during the first 2 Frames, there was no statistical difference between HW and PW in all Frames. HW, Hydrogen rich water; PW, Placebo water. (*p < 0.05 vs Frame 1, N = 10). **(C) Changes in mean power frequency (MPF) every 20 repetitions (rep = 1 Frame) during 100 maximum isokinetic knee extensions.** There was no statistical difference between HW and PW in all Frames. HW, Hydrogen rich water; PW, Placebo water. (*p < 0.05 vs Frame 1, N = 10).

At analysis for maximal knee extension exercise, we divided into five frames of 100-repetition knee extension at the peak torque of isokinetic knee extension exercise [18]. Each frame was corresponded to 20 repetitions; Frame 1 for the first 20 repetitions, Frame 2 for the following 21-40 repetitions, Frame 3 for 41-60 repetitions, Frame 4 for 61-80 repetitions and Frame 5 for the last 81-100 repetitions. Although the peak torque of subjects treated with PW significantly decreased during the first 40 repetitions (Frame 1-2), the reduction of peak torque in the subjects given HW did not reach statistical difference, suggesting that HW inhibited the early decrease of peak torque of the subjects (Figure 2 A).

### MDF and MPF from EMG analysis

MDF and MPF in the subjects treated with PW or HW significantly decreased with time during exercise. While these values significantly decreased at Frame 1-2, there was no statistical difference between the subjects receiving PW and those receiving HW (Figure 2 B, C).

## Discussion

In this preliminary study, we showed that hydration with HW attenuated increase of blood lactate levels and prevented post-exercise decrease of peak torque, an indicator of muscle fatigue. Muscle fatigue is caused by many different mechanisms, including the accumulation of metabolites within muscle fibers and the generation of an inadequate motor command in the motor cortex. The accumulations of potassium, lactate, and H^+^ have often been suggested as being responsible for the decrease in muscle contractility [19]. In addition, aerobic, anaerobic, or mixed exercise causes enhanced ROS production, resulting in inflammation and cellular damage [20]. Short bursts of heavy exercise may induce oxidative stress through various pathways such as electron leakage within mitochondria, auto-oxidation of the catecholamine, NADPH activity, or ischemia/reperfusion [21]. Although the mechanism involved in the efficacies of HW remains unclear, our results show that hydration with HW could be feasible for acute exercise. Proper and adequate hydration is helpful for elite athletes to achieve the best performance. HW can easily replace regular drinking water on a routine basis and would potentially prevent adverse effects associated with heavy exercise.

Factors such as age, nutritional status, training level, and physical activity category can influence the results [22,23]. Although we had anticipated that hydrogen, a known antioxidant, would reduce oxidative stress following acute exercise, the effects of oral intake of HW were marginal and did not affect the level of oxidative markers after exercise. This can be explained by the facts that the athletes in our study have routinely trained and their antioxidant defense systems may be more active. Previous studies reported that repeated aerobic training increases antioxidant enzyme activity and subsequently decreases oxidative stress [2,24-26]. Also, considering the short life-span of hydrogen in circulation [27], more frequent drinking of HW during exercise might have additional effects. In a future study, the efficacy of HW on untrained subjects or recreational exercisers, who may have poorly established antioxidant systems to combat exercise-induced oxidative stress, should be tested. Furthermore, different drinking protocols should be investigated.

We quantified muscle fatigue as a decline in the maximal force or power capacity of muscle, which means that submaximal contractions can be sustained after the onset of muscle fatigue. Similarly, blood lactate concentration is one of the most often measured parameters during clinical exercise testing, as well as during performance testing of athletes. Lactate has often been considered one of the major causes of both fatigue during exercise and post-exercise muscle soreness. Lactate generated from the anaerobic breakdown of glycogen in the muscle occurs only during short bouts of relatively high intensity exercise and it is usually related to fatigue and muscle soreness. Previous evidence has shown that inorganic phosphate from creatine phosphate was the main cause of muscle fatigue [28].

Dehydration in athletes may also lead to fatigue, poor performance, decreased coordination, and muscle cramping. Although further investigations will be warranted, drinking HW may be an appropriate hydration strategy [29]. In this study, we administered HW or PW to subjects prior to exercise. Further investigation is required to determine the best timing, dose, and hydrogen concentration of drinking water to optimize the effects of HW.

In conclusion, our preliminary data demonstrated that consumption of HW reduced blood lactate levels and improved muscle fatigue after acute exercise. Although further studies are absolutely warranted, drinking HW would be a novel and effective fluid hydration strategy for athletes.

## Competing interests

The authors declare that they have no competing interests.

## Authors’ contributions

KA, TA and YM participated in the protocol design and the data accumulation. AN conceived the study and drafted the manuscript. SM participated in the study design and coordination. All authors read and approved the final manuscript.
